# Intracerebral haemorrhage in multiple sclerosis: assessing the impact of disease-modifying medications

**DOI:** 10.1186/s40001-024-01945-x

**Published:** 2024-06-25

**Authors:** Brian M. Ou Yong, Wireko Andrew Awuah, Muhammad Hamza Shah, Vivek Sanker, Jonathan Kong Sing Huk, Sujashree Yadala Venkata, Diti H. Patel, Joecelyn Kirani Tan, Noor Ayman Khan, Ajitha Kulasekaran, Manali Sarkar, Toufik Abdul-Rahman, Oday Atallah

**Affiliations:** 1 School of Medicine, University of Glasgow, Glasgow, UK; 2 Faculty of Medicine, Sumy State University, Sumy, 40007 Ukraine; 3 School of Medicine, Queen’s University Belfast, Belfast, UK; 4 Department of Neurosurgery, Trivandrum Medical College, Thiruvananthapuram, India; 5 Nova Southeastern University Dr. Kiran C Patel College of Allopathic Medicine, Davie, FL USA; 6 Faculty of Medicine, University of St Andrews, St. Andrews, Scotland, UK; 7 DOW Medical College, DOW University of Health Sciences (DUHS), Baba-E-Urdu Road, Karachi, Pakistan; 8 MGM Medical College Navi, Mumbai, Maharashtra India; 9 Department of Neurosurgery, Hannover Medical School, Carl-Neuberg-Strasse 1, 30625 Hannover, Germany

**Keywords:** Multiple sclerosis, Intracerebral haemorrhage, Disease-modifying drugs, Neurological complications, Blood–brain barrier, Neuroinflammation, Pharmacotherapy in MS, CNS immunity

## Abstract

Multiple Sclerosis (MS) is a complex autoimmune disorder that significantly impacts the central nervous system, leading to a range of complications. While intracranial haemorrhage (ICH) is a rare but highly morbid complication, more common CNS complications include progressive multifocal leukoencephalopathy (PML) and other CNS infections. This severe form of stroke, known for its high morbidity and mortality rates, presents a critical challenge in the management of MS. The use of disease-modifying drugs (DMDs) in treating MS introduces a nuanced aspect to patient care, with certain medications like Dimethyl Fumarate and Fingolimod showing potential in reducing the risk of ICH, while others such as Alemtuzumab and Mitoxantrone are associated with an increased risk. Understanding the intricate relationship between these DMDs, the pathophysiological mechanisms of ICH, and the individualised aspects of each patient's condition is paramount. Factors such as genetic predispositions, existing comorbidities, and lifestyle choices play a crucial role in tailoring treatment approaches, emphasising the importance of a personalised, vigilant therapeutic strategy. The necessity for ongoing and detailed research cannot be overstated. It is crucial to explore the long-term effects of DMDs on ICH occurrence and prognosis in MS patients, aiming to refine clinical practices and promote patient-centric, informed therapeutic decisions. This approach ensures that the management of MS is not only comprehensive but also adaptable to the evolving understanding of the disease and its treatments.

## Introduction

Multiple sclerosis (MS) is a chronic autoimmune inflammatory disorder of the central nervous system (CNS) that can lead to neurological defects and severe incapacitation [1]. With a global prevalence of 2.8 million, it is the most common debilitating neurological disease among young adults, with symptoms beginning around the ages of 20–40 [2, 3]. Notably, MS is a challenging diagnosis, as symptoms may vary depending on the severity of the inflammatory reaction as well as the location of CNS lesions. Possible neurological symptoms include vision impairment, focal weakness, paraesthesia, incontinence, and cognitive dysfunction. In addition to the diversity of symptoms, it is common for MS patients to have other comorbid conditions, such as autoimmune conditions like inflammatory bowel disease and rheumatoid arthritis, and less commonly, strokes [4].

Intracerebral haemorrhage (ICH), characterised by bleeding into the brain parenchyma, is the second most common cause of strokes [5]. Spontaneous cerebrovascular haemorrhage has a poor prognosis, with approximately 50% of patients dying within 1 year, and cerebral amyloid angiopathy being the most common cause [6]. The most common cause of ICH is hypertension, but secondary causes include vascular malformations, aneurysms, chronic alcohol use, and medications that increase the risk of bleeding, such as warfarin and apixaban [7]. Risk factors such as hypertension (HTN), hyperlipidemia, and tobacco exposure are suggested to accelerate the progression of MS. However, these vascular risk factors are also tied to cerebrovascular diseases such as ICH. Compared to the general population, MS patients are at an increased risk of developing strokes [8]. A recent retrospective cohort study showed that 0.19% of MS patients experienced ICH. To put this in perspective, the general population experiences ICH at a rate of 24.6 per 100,000 people each year, and it often leads to death [9, 10]. It is worth noting that MS patients face a higher risk of hemorrhagic strokes [8], which adds to the overall severity of their condition. This underscores the importance of prevention strategies, especially since there are limited treatment options available.

Recent MS treatments consist of DMD therapies, which are medications targeted to prevent relapses and progression to disability. This narrative review aims to synthesise the available evidence in the literature, highlighting the influence of various DMDs on the occurrence of ICH in MS patients and exploring their potential role in preventing such occurrences. The research delves into the anti-inflammatory properties of commonly prescribed DMDs in MS management, examining how they may either contribute to or deter ICH. Additionally, this review discusses current practices and future directions to address the research gap, proposing strategies for incorporation into upcoming clinical practices.

## Methodology

**Fig. 1:**
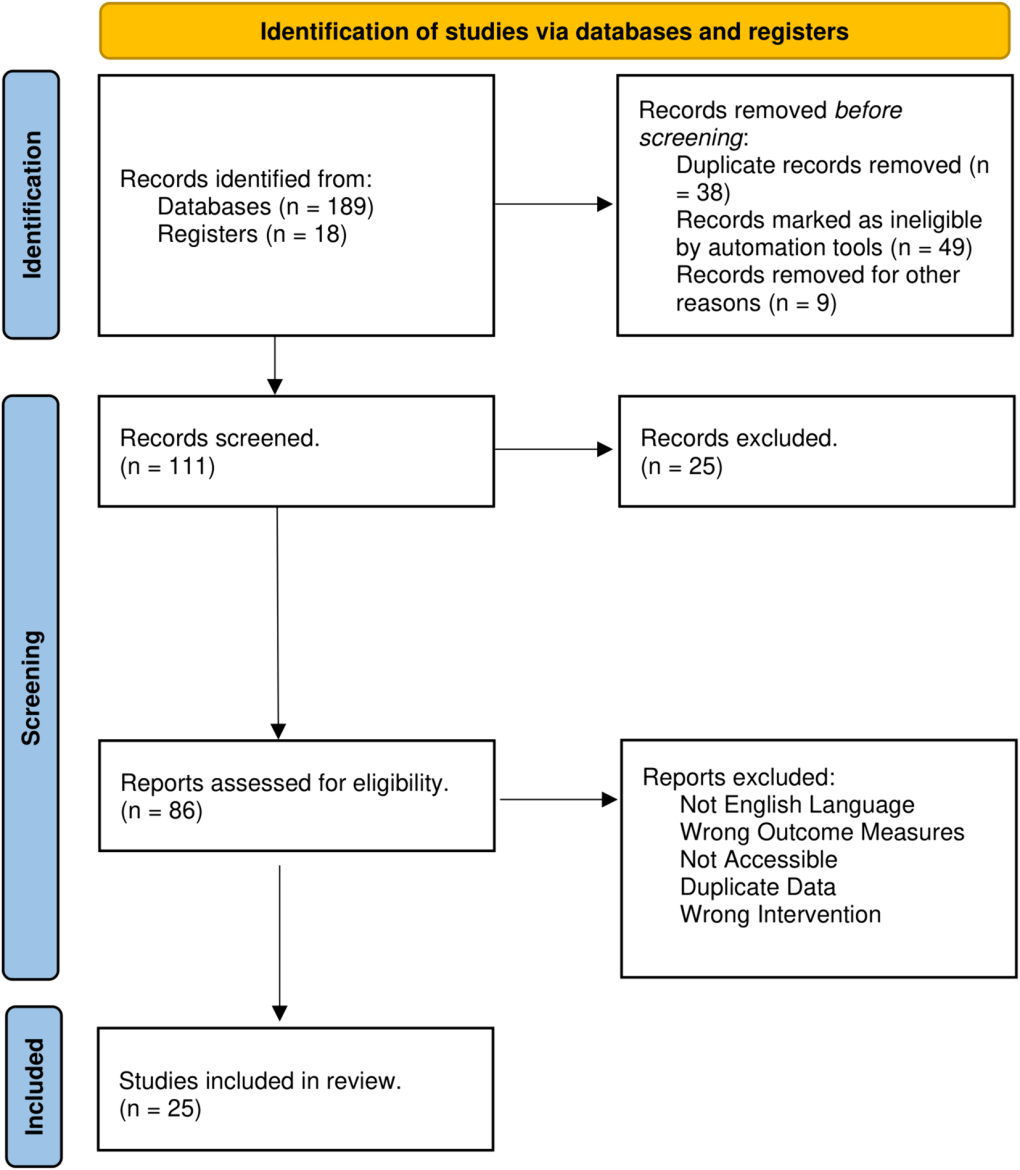
Prisma flow diagram

This narrative review focused on the occurrence of ICH associated with DMDs and their potential protective effect of DMDs against ICH in MS patients. A comprehensive literature search was systematically conducted using PubMed, EMBASE, Google Scholar, and the Cochrane Library, focusing on English-language studies with no timeline applied, encompassing randomised clinical trials (RCTs), meta-analyses, systematic reviews, observational studies, and case–control. Search terms like "disease-modifying drugs," "multiple sclerosis," and “intracranial haemorrhage” were used. A total of 25 articles were included out of 86 articles identified. Furthermore, a manual review of selected articles, reviews, meta-analyses, and practice guidelines was conducted. Abstracts and unpublished studies were excluded from the review. A summary of the methodology employed is presented in Fig. 1.

## Pathophysiology of ICH in MS

The integrity of the blood–brain barrier (BBB) is crucial for maintaining CNS equilibrium. However, in MS, inflammatory autoimmune responses break down the BBB, marked by the recruitment of lymphocytes, microglia, and macrophages to lesion sites [11]. This exacerbates BBB permeability and facilitates further immune cell infiltration, fuelling the inflammatory cascade. Consequently, leukocyte infiltration into the CNS modifies BBB permeability and induces inflammation by expressing inflammatory cytokines, reactive oxygen species (ROS), and enzymes [11]. Inflammatory mediators significantly affect BBB integrity and the immune response. For instance, ICAM-1, stimulated by cytokines like TNF-α, is an early marker of immune activation, correlating with BBB damage, cerebrospinal fluid (CSF) pleocytosis, and TNF-α levels in active MS [12]. Moreover, viruses and environmental pollutants can diminish immunity in genetically susceptible individuals and trigger the release of proinflammatory mediators such as IL-6 and NF-κB [13]. These factors accelerate changes in endothelial tight junctions, increasing BBB permeability and allowing leukocyte migration into the brain parenchyma. This disruption of the BBB plays a critical role in the pathophysiology of ICH in MS patients.

Chronic inflammation in MS can also induce angiogenesis. While angiogenesis is a natural response to tissue injury and inflammation, in the context of MS, it could form abnormal and fragile blood vessels [14]. These newly formed vessels often lack the structural integrity of normal vasculature, making them more prone to rupture. Additionally, the process of vascular remodelling in MS includes the thickening and stiffening of existing vessel walls due to fibrosis [15]. This, combined with the disruption of the BBB, could result in increased vascular resistance and hypertension within the CNS. Both conditions are recognised risk factors for haemorrhage, as the heightened pressure and structural weakness make the vessels more susceptible to rupture under stress.

**Fig. 2:**
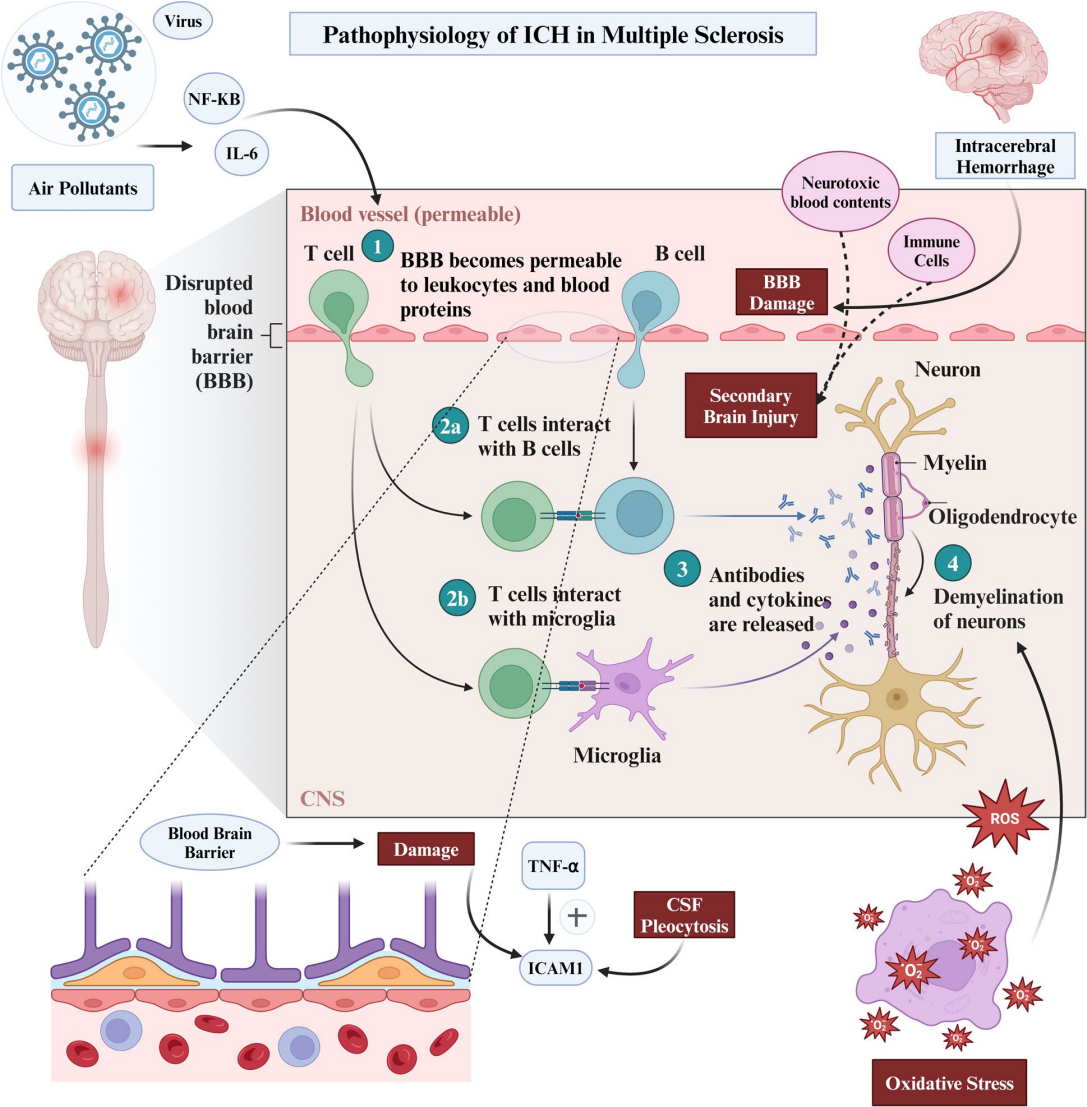
Pathophysiology of Intracerebral Haemorrhage in Multiple Sclerosis Patients. *ICH* Intracerebral haemorrhage, *BBB* Blood–brain barrier, *CSF* Cerebrospinal fluid, ICAM-1 Intercellular adhesion molecule 1, *TNF-α* Tumour necrosis factor alpha, T cells: T Lymphocytes, B cells: B Lymphocytes, *ROS* Reactive oxygen species, *IL-6* Interleukin 6, *NF-κB* Nuclear factor kappa-light-chain-enhancer of activated B cells

Furthermore, vasculitis could also occur in the context of MS [15]. This condition further exacerbates the risk of ICH by weakening the structural integrity of the blood vessels. Inflammatory vasculitis involves immune-mediated damage to the blood vessel walls, which could lead to their thinning and increased fragility [16]. The compromised vessels are at a higher risk of rupture, especially in the dynamic environment of the CNS where blood flow and pressure can fluctuate significantly. As a result, vasculitis in MS patients can create a direct pathway to haemorrhagic events, compounding the already elevated risks due to other inflammatory and structural changes in the vasculature. Figure 2 summarises the complex pathophysiology of ICH in patients with MS.

## Role of DMDs on ICH in MS patients

Various DMDs have been used in the treatment of MS, including Interferon (IFN) beta, Fingolimod, Alemtuzumab, Mitoxantrone (MX), Natalizumab, Siponimod, Dimethyl fumarate, and Ozanimod. Research indicates that certain DMDs, such as dimethyl fumarate, ozanimod, fingolimod, and siponimod, have been associated with a lower risk of ICH in MS patients. Conversely, DMDs like Alemtuzumab, Mitoxantrone, Natalizumab, and IFN-beta have been associated with increased risk of ICH in individuals with MS.

Other alternative DMDs used to treat MS, such as Glatiramer acetate, Daclizumab, Teriflunomide, Fingolimod, Rituximab, Siponimod, Dimethyl Fumarate, and Ocrelizumab, have adverse effects, but there have been no recorded incidences of ICH in MS patients. Some of these drugs have been associated with bleeding issues, but notably, no intracranial bleeding has been reported. For example, Glatiramer acetate is an immunomodulatory drug for treating RRMS. One report describes a case where a patient developed refractory immune thrombocytopenic purpura (ITP) 2 months after starting Glatiramer acetate for MS [17]. During her hospital stay, the patient experienced transient episodes of vaginal, oral, skin, and gastrointestinal bleeding.

### DMDs with potential protective effect against ICH in MS patients

The anti-inflammatory properties of DMDs have been shown to reduce the occurrence of certain diseases, particularly the occurrence of ICH in MS patients, through various mechanisms. A retrospective cohort study done by Zulfiqar et al. showed that DMD could have a protective effect against ICH in MS patients. This effect was shown to persist even after adjusted analyses for potential confounders like lifestyle factors and comorbidities [9]. An in-depth review of the mechanisms of action of the various DMDs will allow us to understand the neuroprotective and neuro-damaging properties of these drugs.

#### Dimethyl fumarate

In a mouse model study, Dimethyl fumarate (DMF) has shown potential in treating ICH in MS through mechanisms involving the activation of the nuclear factor erythroid 2-related factor 2 (Nrf2) pathway. Central to this mechanism is the nuclear factor erythroid-2-related factor 2 (Nrf2), a transcription factor that oversees the expression of antioxidant response element (ARE) genes [18, 19]. In the human brain, Nrf2 is mainly found in non-neuronal cells like microglia and macrophages. The activation of Nrf2 in these cells, particularly through agents like sulforaphane, is noted for enhancing erythrocyte phagocytosis, contributing to the clearance of hematomas [20]. A study involving a group of 26 individuals with ICH indicated the presence of Nrf2 activation in the brain. Although the levels of Nrf2 activation in ICH patients were lower compared to those in the control group, this points to a potential therapeutic avenue for ICH treatment [21].

Further experimental research on ICH in mouse models has connected the anti-inflammatory and neurological improvement properties of DMF to the activation of Casein kinase 2 and the upregulation of Nrf2 signalling pathways [22]. This pathway includes the upregulation of antioxidant genes that protect cells from oxidative damage, which is crucial for neuroprotection in MS patients. Additional studies reinforce DMF's therapeutic potential in ICH scenarios. It has been observed in rat and mouse models that DMF, even when administered 24 h after the onset of ICH, can effectively promote hematoma resolution, reduce neurological deficits, and decrease brain edema, primarily through the activation of Nrf2 genes [23]. Especially notable is the finding that high doses of DMF (100 mg/kg) led to a significant reduction in the brain's fluid content, particularly affecting the ganglia and cortex [23]. These insights highlight the prospective role of DMF in managing ICH for MS patients, largely via mechanisms involving the Nrf2 pathway.

#### Fingolimod

Fingolimod is a sphingosine 1-phosphate receptor (S1PR) modulator that binds to S1PR1, 3, 4, and 5. S1PR1 is expressed in lymphocytes, neurons, glia, and vascular endothelia [24]. It also hinders the regression of lymphocytes from lymph nodes and their recirculation, thereby reducing the migration of pathogenic cells in the CNS. Fingolimod can cross the BBB and enter the CNS to have direct effects on the neural and glial cells. Therefore, through modulation of S1PR in the CNS and immune system, fingolimod has anti-inflammatory and neuroprotective effects in ICH [25]. A study using mouse and rat models to investigate ICH demonstrated that the administration of Fingolimod yielded numerous advantages in ICH management. These benefits encompassed the reduction of brain oedema, short-term enhancements in sensorimotor functions, improved long-term motor coordination and cognitive function, decreased circulating lymphocytes, diminished migration of T lymphocytes into the brain, lowered expression of pro-inflammatory mediators in the brain, and mitigated risk of brain atrophy [26]. In a separate study, it was observed that Fingolimod was well-tolerated by patients with small and moderate-sized ICH in the basal ganglia, leading to improved outcomes and reduced perihaematomal oedema. The compound was found to be beneficial in minimising short-term neurological deficits and promoting enhanced neurologic recovery in the long term [25]. Given that this study was conducted in patients without MS, further investigations are needed to assess its applicability and efficacy in MS patients.

It is evident that Fingolimod holds the potential to exert significant therapeutic effects and offer holistic management of ICH in MS patients. It also has the potential to mitigate the damage caused by hemorrhagic events in the brain. Its ability to inhibit the circulation and migration of pathogenic cells into the CNS could lead to a reduction in the inflammatory response associated with ICH, which may help in limiting secondary damage caused by immune reactions and potentially reduce the risk of recurrent ICH episodes in MS patients. This might contribute to a more stable long-term prognosis [27]. It is worth noting that while S1PR1 plays a pivotal role in mediating the effects of fingolimod, it remains uncertain whether solely targeting S1PR1 is sufficient and indispensable for fingolimod to confer its beneficial effects [27]. Considering the widespread distribution of S1PR1 across various cells involved in the process of ICH, coupled with the preferential localization of RP101075 in the brain, there exists the possibility of other potential cellular targets beyond immune cells and their specific anatomical locations for immune interventions, which necessitates further investigation [27].

Additionally, the activation of *S1PR3* by fingolimod is partially responsible for its undesirable effects on the cardiovascular system and organ fibrosis, potentially posing notable safety concerns. A study has shown that fingolimod can increase blood pressure and predispose patients to stroke [28]. Moreover, there has been a case where an MS patient treated with fingolimod developed posterior reversible encephalopathy syndrome (PRES), which has also been associated with ICH [29, 30]**.** As a result, in January 2012, the European Medicines Agency (EMA) updated its recommendations regarding the use of fingolimod, particularly in MS patients with a prior history of cerebrovascular issues [31]. They also advised that if a particular patient necessitates fingolimod treatment despite their medical history, it is crucial to conduct comprehensive monitoring. Specifically, cardiac activity should be monitored for a minimum of 6 h following the administration of the initial dose via regular ECG tracings and blood pressure measurements [32]. This guidance was further corroborated and adopted by the Central and East European (CEE) MS expert group.

#### Siponimod

Siponimod (BAF312) functions as an S1P analogue, selectively targeting S1PR types 1 and 5, similar to Fingolimod [33]. Studies in a mouse model of ICH have demonstrated Siponimod’s multiple benefits, including reduced lymphocyte counts leading to lymphopenia, decreased brain and perihaematomal edema, improved survival rates, better neurological outcomes up to 72 h post-ICH, and reduced weight loss [33]. These effects are thought to stem from siponimod’s capacity to modulate brain tissue inflammation through S1PR1, thereby limiting secondary brain damage [33]. Siponimod operates by making S1PR1 receptors unresponsive to normal exit signals from lymph nodes [34] and exerts other actions such as affecting glial cell function, reducing demyelination, and lowering circulating monocyte levels, independently of S1PR3 [35]. However, siponimod also activates G-protein-coupled inwardly rectifying potassium channels in human atrial myocytes, potentially explaining the observed rapid yet temporary bradycardia onset in some studies [34]. Additionally, its interaction with 5-G protein-coupled S1P receptors, located in crucial organs like the lungs, heart, and kidneys, suggests a broader impact on various physiological processes during treatment [34].

The aforementioned studies suggest that the multifaceted approach to siponimod makes it potentially beneficial for treating ICH in MS patients. However, given its impact on various physiological processes and the potential cardiovascular effect of the medication, it is crucial to exercise caution when used in patients, especially those with predisposing factors. There is an imperative need for further research and clinical trials that investigate the use of siponimod in patients with ICH, given the promising results observed in preclinical studies.

#### Ozanimod

**Fig. 3:**
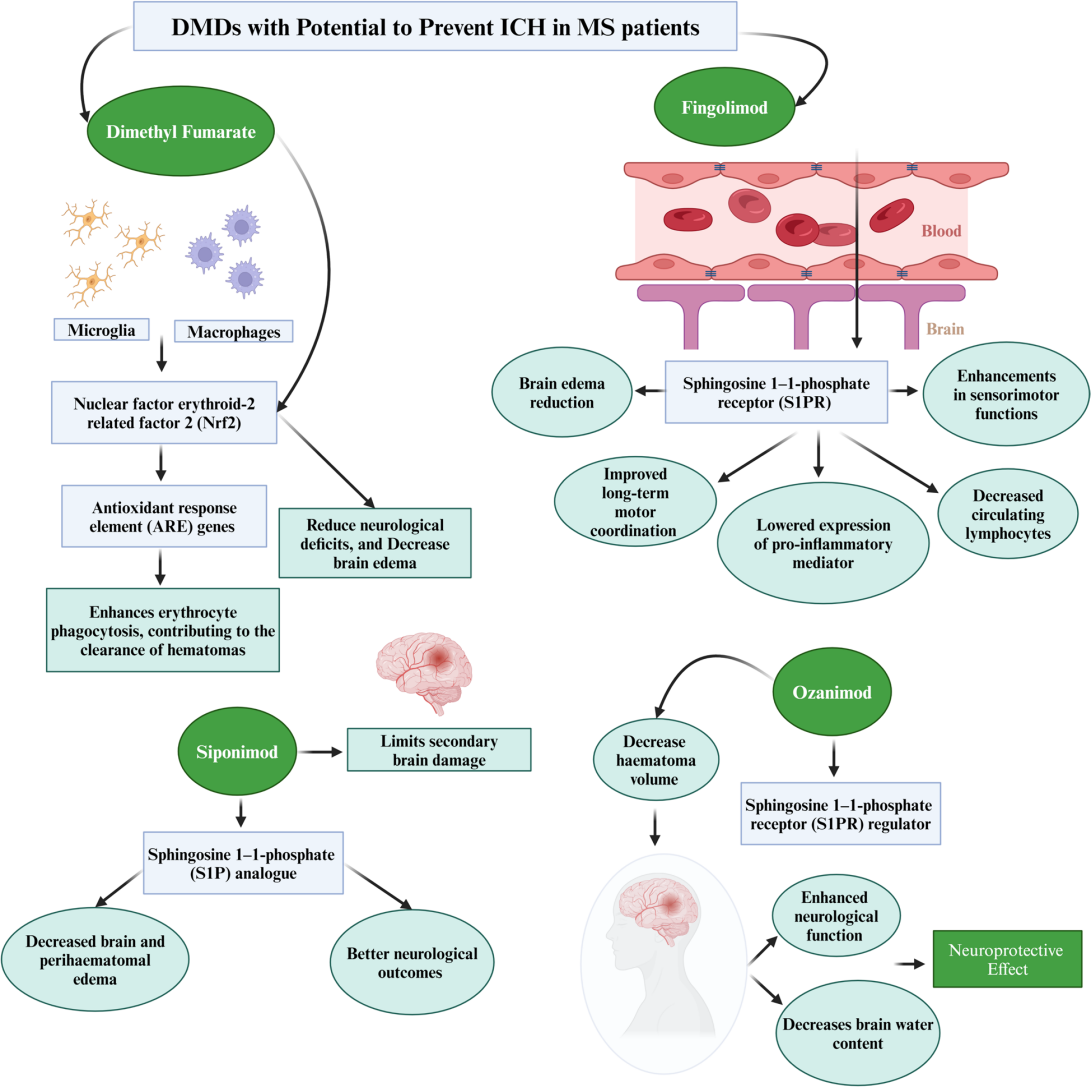
Disease-modifying drugs with protective effect against intracerebral haemorrhage in multiple sclerosis patients. *DMD* Disease-modifying drugs, *ICH* Intracerebral haemorrhage, *MS* Multiple sclerosis, *Nrf2* Nuclear factor erythroid-2-related factor 2, *S1P* Sphingosine 1-1-phosphate, *S1PR* Sphingosine 1-1-phosphate receptor

Ozanimod, an oral sphingosine-1-phosphate (S1P) receptor modulator, has been found to be effective in reducing the annualised relapse rate, new or enlarging T2 lesions, and gadolinium-enhancing lesions in MS patients through selective modulation of S1P1 and S1P5 receptors [36, 37]. In mice with ICH, Ozanimod was found to decrease hematoma volume and subsequent brain water content, leading to enhanced neurological function and reduced body weight loss post-ICH [36]. This effect is attributed to the reduction of activated microglia and infiltrated neutrophils surrounding the hematoma. Additionally, the study also highlighted Ozanimod’s ability to diminish brain cell death and preserve the integrity of the BBB, further emphasising its neuroprotective effects [36]. Ozanimod exhibits a strong preference for the S1PR1 subtype over S1PR5. Its selectivity for S1PR1 is over 10,000 times greater than for S1PR2, 3, and 4 [37]. This high specificity of Ozanimod for S1PR1 helps reduce potential safety concerns related to the activation of S1PR3, which has been associated with various adverse events including hypertension, macular oedema, pulmonary toxicity, and liver toxicity [38]. Given these findings, the neuroprotective effects observed in mouse models of ICH can be considered relevant for MS patients, where Ozanimod’s mechanism of reducing activated microglia and infiltrated neutrophils, decreasing hematoma volume, and preserving the integrity of the blood–brain barrier could potentially translate into protective effects against CNS injuries, including ICH. DMDs with the potential to prevent ICH in MS patients are illustrated in Fig. 3.

### DMDs associated with increased ICH risk in MS patients

DMDs play a crucial role in managing MS by altering the disease course and reducing relapse rates. However, the broad effects of DMDs, especially concerning ICH, necessitate a detailed understanding of their impacts.

#### Alemtuzumab

Alemtuzumab is a monoclonal antibody that targets the CD52 protein on T and B lymphocytes, effectively reducing inflammation. Notably, there have been reports of ICH in patients treated with alemtuzumab. For instance, a study documented five instances of ICH in patients with relapsing–remitting multiple sclerosis (RRMS) after they received only three to five doses of alemtuzumab [39]. These cases of ICH occurred within hours of the infusion, and notably, these patients had no previous history of bleeding. Symptoms like headaches and chest pain were reported during the drug infusion [39]. In another case, a patient developed ICH and subsequently passed away 6 days after starting an intravenous infusion of alemtuzumab, displaying symptoms such as headache, vomiting, and bradycardia [40]. Furthermore, a significant decrease in the patient's platelet count was noted on the fifth day after starting the treatment [40]. These cases highlight the potential risk of ICH in patients undergoing treatment with alemtuzumab, underscoring the need for careful monitoring, especially in the initial stages of treatment.

The exact mechanism through which alemtuzumab leads to ICH remains uncertain, but one theory points to the drug's potential effect on blood pressure. Research has shown that there's an average increase in mean systolic and diastolic blood pressure (SBP and DBP) of about 20 mmHg and 6 mmHg, respectively, after the first alemtuzumab infusion [41]. Moreover, studies have highlighted a correlation between alemtuzumab administration and blood pressure changes or increases, occurring even beyond the typical monitoring period after administration [39]. This suggests a potential link between elevated SBP and DBP and an increased risk of ICH. Contrarily, another study found no significant difference in mean SBP following alemtuzumab infusion [42]. Consequently, revised recommendations during alemtuzumab administration include considering inpatient admission for MS patients on alemtuzumab whose mean systolic blood pressure increases significantly during infusion or those who have a notable rise above their baseline due to the ICH risk [43]. In such cases, thorough monitoring of vital signs, frequent neurological assessments, and strict blood pressure control are advised during the hospital stay [39]. If the patient can tolerate a different DMD, considering a switch from alemtuzumab is recommended, and these guidelines have been incorporated into the American MS DMD guidelines. Before administering alemtuzumab, a detailed review of the patient's medical history, including any bleeding disorders or strokes, current medication, blood pressure readings, platelet counts, and risk factors for developing ICH, is crucial for personalised treatment decisions and risk assessment.

In addition to the potential blood pressure-related mechanisms, alemtuzumab may also induce ICH through secondary immune thrombocytopenic purpura (ITP). Reports indicate that 1.54% of patients with ITP developed ICH as a complication [44]. A patient was documented as developing drug-induced ITP after alemtuzumab treatment. Further supporting this, an analysis of alemtuzumab patients revealed that 2.3% developed ITP [45]. While the exact mechanism is unknown, the association between alemtuzumab and ICH is underscored by the connection between alemtuzumab-induced ITP and the occurrence of ICH.

#### Mitoxantrone

Mitoxantrone (MX) is recognized for its immunosuppressive properties, particularly in the context of MS, where it plays a crucial role in inhibiting the proliferation of T cells, B cells, and macrophages, reducing antigen presentation, and diminishing the release of proinflammatory cytokines [46]. While incidents of ICH related to MX usage are rare, a case was reported where a patient developed ICH after being diagnosed with acute myeloid leukaemia (AML) after being administered a single MX dose. Notably, a blood test performed two days prior to the ICH diagnosis showed a significantly reduced platelet count in the patient [47]. The mechanism by which MX might lead to an increased risk of ICH in MS patients can be traced back to its known side effects, which include the potential to induce TRAL. A study reviewing 12511 patients on MX found a minor percentage developing TRAL, with a significant portion of these cases being acute promyelocytic leukaemia (APL) and acute myelocytic leukaemia (AML), both of which can lead to thrombocytopenia [48]. Additionally, there was a report of a patient who developed therapy-related pre-B cell acute lymphoblastic leukaemia and pancytopenia only 6 months after starting MX treatment [49]. The precise mechanism through which MX induces ICH is not well defined. However, it is recognised that ICH is a common complication in leukaemia cases, especially when accompanied by thrombocytopenia—a condition frequently observed in 40–60% of leukaemia patients [50, 51]. The connection between thrombocytopenia and the incidence of ICH in patients with leukaemia has been well documented [50]. Therefore, it is plausible to consider that MX might lead to ICH by first inducing TRAL, accompanied by thrombocytopenia, which in turn could lead to the development of ICH.

#### Natalizumab

Natalizumab, a monoclonal antibody, functions by hindering leukocyte adherence to endothelial cells through the blockade of the α4-integrin subunit, which is found in lymphocytes, monocytes, and eosinophils [52]. By interrupting this interaction, Natalizumab prevents leukocytes from migrating into the target organ, thereby effectively reducing inflammation [52]. A case was reported suggesting a possible association between Natalizumab and ICH. In this case, a patient developed ICH 22 days following the third dose of Natalizumab, despite having no prior history of bleeding disorders, vascular complications, or hypertension [53]. Given that only one case has been reported, it is crucial to exercise caution in suggesting that Natalizumab can predispose to ICH. One proposed mechanism for Natalizumab-induced ICH is its potential effect on angiogenesis inhibition. Research has shown that α4β1-integrin plays a role in angiogenesis by facilitating the adhesion of large-vessel endothelial cells to the extracellular matrix proteins thrombospondin 1 (TSP1) and thrombospondin 2 (TSP2). Inhibition of α4β1 can disrupt angiogenic processes [54]. TSP1 and TSP2 are also known to support endothelial cell survival and proliferation via α4β1-integrins [54]. It has been observed that neutralising antibodies against endothelial α4-integrin significantly hamper angiogenesis triggered by factors like tumour necrosis factor-α and soluble VCAM-1 [55]. Additionally, α4β1-integrin is instrumental in the homing of CD34 + progenitor cells to the vascular endothelium during the process of neovascularization, which is critical for tissue repair [56]. Consequently, the inhibition of α4-integrin-mediated angiogenesis presents a plausible hypothesis for the occurrence of haemorrhage.

#### IFN-β

**Fig. 4:**
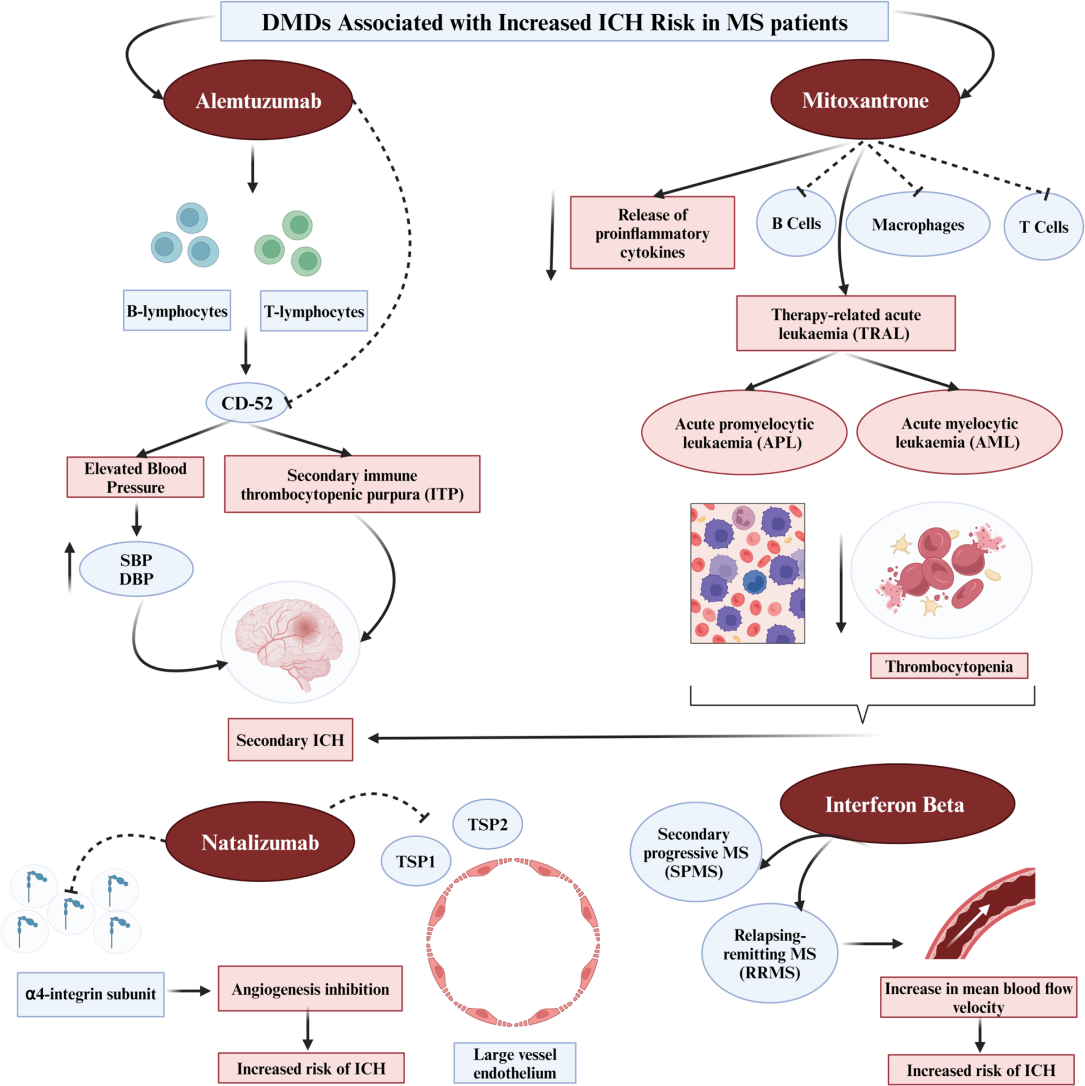
Disease-modifying drugs associated with increased ICH risk in MS patients. Abbreviations: *AML* Acute myelocytic leukaemia, *APL* Acute promyelocytic leukaemia, *CD-52* Cluster of differentiation-52, *DBP* Diastolic blood pressure, DMD Disease-modifying drugs, *ICH* Intracerebral haemorrhage, *ITP* Immune thrombocytopenic purpura, *MS* Multiple sclerosis, *RRMS* Relapsing–remitting multiple sclerosis, *SBP* Systolic blood pressure, *SPMS* Secondary progressive multiple sclerosis, *TRAL* Therapy-related acute leukaemia, *TSP* Thrombospondin

IFN-β has a multifaceted mechanism of action that is not completely understood. It seems to augment the levels and expression of anti-inflammatory substances while concurrently decreasing the expression of proinflammatory cytokines [57]. IFN-β engages with specific receptors on human cell surfaces, setting off a cascade of events leading to the expression of various interferon-stimulated genes and markers such as MHC Class I, Mx protein, OAS, β2-microglobulin, and neopterin [57]. Different forms of IFN-β, including IFN-β-1a, IFN-β-1b, and peginterferon beta-1a, are commonly used to manage MS, each having similar mechanisms of action and effectiveness but varying in terms of administration routes and tolerability [58]. Despite its prevalent use and typically mild to moderate side effects, the potential of IFN-β to cause ICH has been underexplored [59]. Cases have been reported where patients with prolonged IFN-β treatment, specifically those with secondary progressive MS (SPMS) and relapsing–remitting MS (RRMS), experienced sudden symptoms leading to the discovery of substantial ICH, even in the absence of prior conditions like hypertension, headache, or thrombocytopenia [60, 61]. While IFN-β is generally perceived as safe, there have been occurrences of unforeseen adverse effects, including unreported instances of ICH. The exact mechanism connecting IFN-β to ICH is still speculative, but it is proposed that extended use may induce vascular changes, subsequently increasing the ICH risk [60]. Investigations, such as a pilot study examining IFN-β-1a's impact on intracranial vascular tone regulation in RRMS patients, have shown significant changes, like an increase in mean blood flow velocity in several cerebral arteries 10 h after IFN-β administration [62]. These findings suggest a potential association between IFN-β administration and unrecognised vascular modifications that might predispose individuals to ICH. DMDs associated with increased ICH risk in MS patients are illustrated in Fig. 4.

## Discussion and future prospects

Recent research into the impact of DMDs on ICH in MS has highlighted the multifaceted nature of these medications. Studies have reinforced the role of DMDs in reducing disability progression in MS, showcasing their significant role in managing the disease. However, their broader impacts, particularly concerning ICH, require careful consideration due to the varying effects on the immune system and other physiological processes [63]. The differential impact of DMDs on peripheral blood B cell subsets has been noted, which may influence their overall effect on the immune system. This differentiation is crucial in understanding the comprehensive effects of DMDs, including their potential implications for ICH risk and severity [64].

Moreover, the possibility of repurposing drugs initially developed for MS might offer new therapeutic pathways for ICH management. However, rigorous preclinical and clinical validation is paramount before these drugs can be considered for clinical use in the context of ICH [65]. The safety profiles of DMDs are under constant scrutiny, with ongoing pharmacovigilance studies being pivotal in understanding the adverse drug reactions (ADRs) associated with these therapies. This knowledge is crucial for optimising the use of DMDs and minimising potential risks, including ICH [66].

Future prospects involve a comprehensive understanding of the molecular pathways influenced by DMDs, the identification of patient-specific factors influencing drug efficacy and safety, and the development of rigorous monitoring protocols to mitigate risks. Large-scale, multicentric longitudinal studies are crucial to gathering robust data on the long-term effects of DMDs on the incidence and prognosis of ICH in MS patients. This includes understanding the molecular pathways influenced by these drugs, identifying patient-specific factors influencing drug efficacy and safety, and developing rigorous monitoring protocols to mitigate risks [67]. Emerging therapies that modulate neuroinflammation hold promise for improving the prognosis of ICH. Understanding the role of drugs like minocycline, sphingosine-1-phosphate receptor modulators, and statins in controlling neuroinflammation could revolutionise the treatment landscape for ICH [68]. For instance, CAA, which is one of the common causes of lobar haemorrhage, currently has limited therapeutic options. However, a recent study has shown that minocycline was associated with a reduction in ICH recurrence [69].

Recent studies have voiced the need for therapies that can arrest and reverse the persistent accumulation of disabilities associated with progressive forms of MS. Neural stem cell (NSC) therapies have shown unexpected neurotrophic support and the ability to inhibit detrimental host immune responses following transplantation into the chronically inflamed CNS [70]. Understanding the underlying mechanisms of these therapies and validating their efficacy through clinical trials could open new avenues for MS treatment, especially in its progressive stages. The protracted nature of neuroinflammation in ICH provides a window of opportunity for innovative therapies to subdue the undesired consequences. Investigating the potential of histaminergic drugs in MS and their influence on the differentiation of oligodendrocyte precursors, demyelination, and the remyelination process presents a novel approach to addressing neuroinflammation and fostering repair mechanisms in the CNS [71].

Moreover, new research also highlights the prognostic utility of serum biomarkers such as S100 calcium-binding protein B, white blood cell count, and copeptin, which could potentially guide the selection of DMDs for individual patients, tailoring treatments to mitigate risks while maximising efficacy [72]. Similarly, the differentiation in the impact of short-term versus long-term use of DMDs on the incidence and severity of ICH presents a critical area for future investigation. Understanding the optimal duration of DMD therapy is essential for balancing the therapeutic benefits against potential adverse effects. Moreover, considering demographic factors such as age and gender in the context of DMD treatment can lead to more personalised approaches, as these factors may influence the risk profile for ICH in MS patients [72].

## Study limitations

While this review provides a comprehensive synthesis of existing literature on the impact of DMDs on ICH in MS patients, it is essential to acknowledge certain limitations that may impact the generalisability and depth of the findings. Firstly, the inclusion of studies was limited to those available in the selected databases and relevant literature, potentially leading to a bias in the reviewed evidence. The exclusion of studies published in languages other than English may have resulted in the oversight of relevant contributions from non-English literature. Additionally, the retrospective nature of narrative reviews inherently introduces a risk of selection bias, as the studies included were based on the author's judgement and interpretation of the role of DMDs on ICH in MS patients. Moreover, ICH represents a rare complication in MS. Consequently, the infrequent occurrence of ICH in MS patients results in a scarcity of data, limiting the basis from which findings can be extrapolated. Most of the data currently available are derived from mouse models, with limited data from human studies involving MS patients. This disparity underscores the need for further research specifically focused on human subjects to better understand the potential link between DMDs and ICH in the MS population. Currently, there is inadequate research dedicated to investigating the severity of ICH in MS patients undergoing DMD therapies. It is crucial to explore this aspect, considering the potential influence of lifestyle, socioeconomic factors, and addiction determinants on both the occurrence and severity of ICH in this specific population. Finally, variations in study methodologies and outcome measures across the included studies may contribute to heterogeneity and limit the ability to draw definitive conclusions.

## Conclusion

The review outlines the impact of various DMDs on ICH occurrence and prevention. Noteworthy findings indicate that certain DMDs, such as Dimethyl Fumarate, Fingolimod, Siponimod, and Ozanimod, exhibit potential protective effects against ICH, while others like Alemtuzumab, Mitoxantrone, Natalizumab, and Interferon beta may pose risks. However, given the complexity surrounding MS, DMDs, and ICH, caution is warranted in asserting that these medications definitively prevent ICH in MS patients. Considering the complexity surrounding MS, DMDs, and ICH, this review calls for ongoing research to address the identified limitations. A thorough exploration of the mechanisms underlying DMD effects, coupled with well-designed comparative studies, will contribute to a more nuanced understanding of ICH in the context of MS treatment. These insights can inform clinical practices, enhance patient care, and guide the development of tailored therapeutic approaches in the ever-evolving landscape of MS management.

## Data Availability

No additional data available.

## Abbreviations

MS: Multiple sclerosis
CNS: Central nervous system
ICH: Intracerebral haemorrhage
HTN: Hypertension
DMD: Disease-modifying drug
RCT: Randomised clinical trial
BBB: Blood–brain barrier
CSF: Cerebrospinal fluid
ICAM-1: Intercellular adhesion molecule 1
TNF-α: Tumour necrosis factor alpha
ROS: Reactive oxygen species
IL-6: Interleukin 6
NF-κB: Nuclear factor kappa-light-chain-enhancer of activated B cells
SAH: Subarachnoid haemorrhage
IFN: Interferon
DMF: Dimethyl fumarate
Nrf2: Nuclear factor erythroid-2-related factor 2
ARE: Antioxidant response element
S1PR: Sphingosine 1-phosphate receptor
MX: Mitoxantrone
RRMS: Relapsing–remitting multiple sclerosis
EMA: European Medicines Agency
CEE: Central and East European
PML: Progressive multifocal leukoencephalopathy
AML: Acute myeloid leukaemia
TRAL: Therapy-related acute leukaemia
APL: Acute promyelocytic leukaemia
SPMS: Secondary progressive multiple sclerosis
SBP: Systolic blood pressure
DBP: Diastolic blood pressure
ITP: Immune thrombocytopenic purpura
TSP1: Thrombospondin 1
TSP2: Thrombospondin 2
VCAM-1: Vascular cell adhesion molecule 1
MHC: Major histocompatibility complex
CIS: Clinically isolated syndrome
NSC: Neural stem cell
CNS: Central nervous system
